# Influence of photobiomodulation therapy on regenerative potential of non-vital mature permanent teeth in healthy canine dogs

**DOI:** 10.1007/s40368-025-01000-1

**Published:** 2025-01-31

**Authors:** S. F. Khattab, Y. F. Gomaa, E. A. E. Abdelaziz, N. M. A. Khattab

**Affiliations:** 1 Ministry of Interior, Cairo, Egypt; 2 Faculty of Dentistry, Minia University, El Minia, Egypt; 3 Faculty of Dentistry, Ain Shams University, Cairo, Egypt

**Keywords:** Blood clot scaffold, Double antibiotic paste, Mature dog teeth, Photobiomodulation therapy, Regenerative endodontic procedures, Animal models

## Abstract

**Purpose:**

The purpose of this study is to evaluate the influence of photobiomodulation therapy on the regenerative potential of non-vital mature permanent teeth of healthy Canine dogs.

**Methods:**

54 mature roots, obtained from 27 premolars, in dogs, were selected and divided into three equal groups where Group I received regenerative endodontic procedures (REPs) using blood clot as a scaffold (positive control), Group II received similar treatment as Group I, followed by photobiomodulation therapy (study group) and Group III did not receive any intervention (negative control). Each group was further divided into three equal subgroups for the evaluation of the vascular area percentage and fibroblast count at 3 different intervals; 3, 10, and 15 weeks. Statistical analysis was performed with R statistical analysis software version 4.1.3

**Results:**

Data analysis for vascular area percentages, revealed an insignificant increase in mean values at 3 and 10 weeks for both the positive control and study group, and all values were significantly lower than the negative control (*p* < 0.001). At 15 weeks, there was an insignificant difference between the study group and the negative control group, with mean values was 9.76 ± 2.25, and 11.97 ± 2.37, respectively. However, both values were significantly higher than the positive control group (2.47 ± 1.0), (*p* < 0.001).

Regarding fibroblast count, there was a gradual increase in mean values recorded at different intervals, reaching its maximum at the 15th week period; they were 41.40 ± 1.14, 56.60 ± 6.11 and 44.67 ± 17.04 for positive control, study group, and negative control respectively, with insignificant differences between the study group and negative control.

**Conclusion:**

The results of this study support the revascularization of non-vital mature permanent teeth as an alternative treatment modality. Photobiomodulation could improve the construction of regenerated blood vessels and fibroblasts. However, further studies with longer flow-up periods and different animal models are recommended.

## Introduction

Conventional endodontic treatment has been the primary choice for treating traumatised and infected; nonvital permanent teeth for a long time. However, this treatment has been associated with several complications like broken instruments, perforations, over or under-filing, increased risk of tooth fracture during mechanical preparation, as well as failures resulting from the residual microorganisms’ colonies that sometimes do not respond to disinfection protocols (Goldberg 2016; Bhuva and Ikram 2020).

Even in cases following the highest technical standards, sometimes failures occur due to microbial factors, comprising extra-radicular and intra-radicular infection, and intrinsic and extrinsic non-microbial factors (Borisova-Papancheva et al. 2022). The current conventional endodontic treatment is based on the concept of sealing disinfected root canals with as little residual space as possible to minimise bacterial recolonization (Al-Nazhan et al. 2014); however, space elimination for nanometer-sized bacteria by gutta-percha is nearly impossible to achieve. If the dental pulp is regenerated, natural killer cells, lymphocytes, and macrophages may be restored. This creates an innate immune system in the regenerated teeth and becomes structurally more resistant to fracture than endodontically treated teeth (Saghiri et al. 2015).

The Regenerative Endodontic Procedure (REP) has been introduced in the treatment of necrotic immature permanent teeth by creating a bacteria-free environment and introducing stem cells, scaffolds, growth factors, and tight coronal seal. Such therapy has shown satisfactory success rates thus encouraging researchers to evaluate its applicability in the treatment of necrotic mature permanent teeth (Laureys et al. 2013; Chrepa et al. 2015). This treatment circumvents the weakening of the dentine walls by avoiding the step of shaping of dentine root walls. It also eliminates the use of artificial biocompatible filling material which can evoke foreign body reactions that could lead to the persistence of periradicular lesions (Karamifar et al. 2020).

It is worth mentioning that, the idea of regeneration in mature permanent teeth has been supported by the possibility of releasing growth factors from dentine walls by Ethylene Diamine Tetra Acetic acid (EDTA) irrigation during the disinfection process (Farhad et al. 2022), and that, some studies have suggested the possibility of tissue regeneration without scaffold (Syed-Picard et al. 2014; Dissanayaka et al. 2015). Also, histological studies have proven that apical foramen as small as 0.32 mm did not prevent the ingrowth of new tissues (Laureys et al. 2013; Saoud et al. 2016). Moreover, stem cells, and scaffolds which are essential elements for regeneration can be obtained from provoked bleeding. Such bleeding is induced in the canal space by mechanical stimulation of periapical tissues. Cellular expansion could be allowed through growth factors which may be obtained from platelets in blood clots (Alghofaily et al. 2022).

Although previous systematic reviews have shown high clinical success rates after REPs, (Torabinejad et al. 2017; Tong et al. 2017; Kahler et al. 2017) their meta-analyses showed lower radiographic success rates (Tong et al. 2017; He et al. 2017). There are some restrictions concerning REPs related to the number of stem cells that migrate into the root canal system, their proliferation, and survival capabilities that could be threatened in such procedures (Lovelace et al. 2011).

So, Photobiomodulation therapy (PBM) has been proposed to improve the process of tissue regeneration in REPs (Zaccara et al. 2019). PBM is defined as a form of light therapy that utilises non-ionising light sources in the visible and infrared spectrum. Such therapy is considered a non-thermal process involving endogenous chromophores producing photophysical and photochemical actions at various biological scales. It encourages biostimulation effects on different cell types by improving the environmental situations compensating oxidative stress and increasing blood flow to implanted stem cells to enhance their survival rate and avoid their early death (Zaccara et al. 2019; Alnagar et al. 2019).

PBM provides enhancement of cellular metabolism through activation of the cellular respiratory chain of mitochondria allowing changes in redox properties through a primary molecular process that involves a measurable biological effect at the cellular level followed by secondary reactions by cellular signalling and amplification chain occurring in cell cytoplasm, membrane, and nucleus, (Hamblin 2017).

There are five hypotheses tried to clarify the PBM mechanism of action but all of them lead to a similar result. The first hypothesis is the singlet-oxygen hypothesis which suggests that some respiratory-chain components can be reversibly converted to photosensitizer that when photoactivated leads to the generation of singlet oxygen which leads to stimulation of the RNA and the DNA synthesis. The next hypothesis is the redox properties alteration hypothesis which supposes that the photoexcitation of the cytochrome c-oxidase molecule influences its redox state and consequently the rate of electron flow in the molecule. The NO hypothesis hypothesised that laser irradiation and activation of electron flow in the molecule of cytochrome c-oxidase could reverse the partial inhibition of the catalytic centre by NO and in this way increase the oxygen binding and respiration rate. The fourth one superoxide anione hypothesis which depends on the activation of the respiratory chain that increases the production of superoxide anions, and the last one is the transient local heating hypothesis that causes structural changes by local transient rise in temperature of absorbing molecules. (Cronshaw and Mylona 2023).

The development of lasers and their benefits in medical field offered promising ways that can overcome the limitations of REPs and could provide exciting achievements (Arapostathis et al. 2023). For example, the use of Photodynamic Therapy (PDT), which was suggested as a promising effective adjunct to standard antimicrobial intracanal cleaning and shaping, led to a reduction of up to 99% in colony-forming unit counts particularly for teeth undergoing one-session endodontic treatment (Johns et al. 2014). Also, several studies have shown that PBMT can improve cell migration, growth, and proliferation, increase cell metabolism, as well as, improving the synthesis of growth factors, which, in turn, will enhance cell differentiation (Peng et al. 2017; Marques et al. 2017; Diniz et al. 2018).

Regarding the mechanism of activity following PBM therapy, PBM was found to activate redox reactions, which increase the cell–matrix attachment, which has a role in the regulation of gene transcription, so regulates the process of cell growth, differentiation, and morphogenesis. PBM stimulates basic fibroblast growth factor production to support fibroblast proliferation, differentiation, and maturation. PBM has proved to increase the expression of vascular endothelial growth factor, synthesis, and production of angiogenic factors by T-lymphocytes which play a role in angiogenesis which is the essential component of normal development, tissue repair, and regeneration, through their lymphokines that regulate different functions of endothelial cells which form capillaries and line other vessels of microcirculatory bed. In addition, PBM has proved to be a potent dilator of the arterioles, causing a marked increase in blood flow in microvascular bed, and an increased degree of oxygenation of erythrocytes which supports its benefits in regenerative endodontics. (Dipalma et al. 2023).

Despite the increased interest in studying the effect of REPs using blood clots as a scaffold in mature permanent teeth, data about the effects of PBMT as an associated treatment to REPs is insufficient. Considering the positive effect of Photobiomodulation on dental pulp regeneration, the current study was designed to evaluate the influence of photobiomodulation therapy on the regenerative potential of non-vital mature permanent teeth in dogs. The null hypothesis was that no statistically significant difference would be found between the tested groups in terms of vascular area percentage and fibroblast count.

## Materials and methods

The Scientific Research Ethical Committee of the Faculty of Dentistry, Minia University evaluated and approved the experimental proposal of the current study for the animal experiments guidelines and regulations (reference number 264/2019). All procedures were carried out in accordance with NC3Rs (National Centre for the Replacement Refinement and Reduction) of Animals in Research guidelines and regulations. All procedures were reported in accordance with ARRIVE guidelines of animal experiments and revised with PRIASE 2021 guidelines for reporting animal studies in Endodontology.

### Sample size

Sample-size calculation was performed by a power analysis that was designed to have adequate power to apply a statistical test of the null hypothesis that there is no difference would be found between different tested groups. By adopting an alpha level of (0.05) a beta of (0.2) i.e. power = 80% and an effect size (*f*) of (0.435) calculated based on the results of a previous study (Fahmy et al. 2017), the predicted sample size (*n*) was a total of (54) samples. Sample-size calculation was performed using G*Power version 3.1.9.7 (Faul et al. 2007).

### Specimen randomisation and allocation

A total of 27 multi premolars (54 mature roots) were selected from 3 mixed breed canine male dogs about 10–12 months old with weights between 12 and 15 kg. Samples were randomly allocated by an independent investigator (NMAK) using a closed sealed envelope into three equal groups (9 premolars; 18 roots/each) according to the treatment protocol. One side in each dog was allocated to the photobiomodulation group (either upper or lower), whereas the other side was allocated randomly amongst the other two groups.

**Grouping:** Group I (positive control group), was treated with regeneration procedures using a blood clot as a scaffold, Group II (study group), was treated with the same regenerative protocol as Group I, then subjected to photobiomodulation therapy, Group III (negative control group), no intervention has been made and used for evaluation of normal dog’s pulp to be compared with regenerated pulp.

**Fig. 1:**
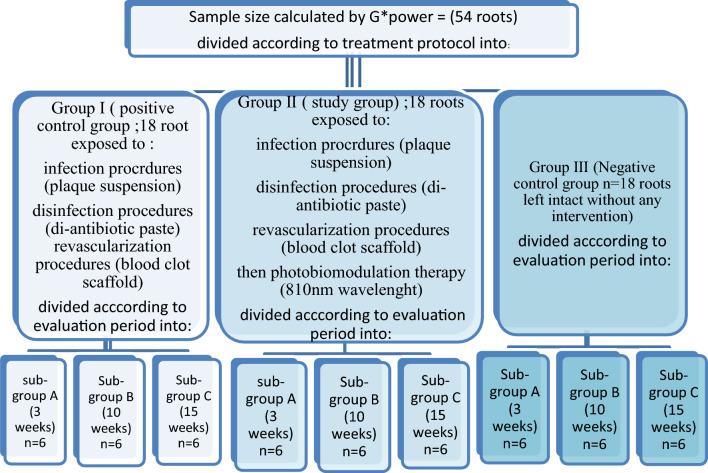
Flowchart illustrating group distribution according to the treatment protocols and evaluation periods for dogs’ teeth

Each group was subdivided into three subgroups (*n* = 6 roots per subgroup) according to the evaluation periods, which were Subgroup A: 3 weeks, Subgroup B: 10 weeks, and Subgroup C: 15 weeks. The study groups and subgroups are illustrated in Fig. 1

### Animal care

Following the National Centre for the three Rs (Replacement Refinement and Reduction) of animals in research and the Canadian Council regulations of animal care, the selected dogs were housed in separate cages located in a room provided with artificial light to offer a stimulated cycle of day and night. The cages were parasite-free, air-dried ventilated, away from the noise and humanoid inhabitation, and had fresh water and a balanced soft diet (Fenwick et al. 2011). They were given 5-way vaccine and booster doses for rabies. The weight of the selected dogs was preserved between 12 and 15 kg (Fahmy et al. 2017).

**Experimental procedures**: were carried out at the Veterinary Unit, General Organization for Veterinary Services. Anaesthesia for dogs was induced intravenously with Xylaject 2% (1.1 mg/kg) intramuscular, Atropine sulphate (0.1 mg/kg) subcutaneous, Thiopental Sodium 2.5% (25 mg/kg) intravenous, and Ketamine Hcl 10% (15 mg/kg) intramuscular. This was followed by Dexamethasone (0.5 mg/kg) and Metacam (2 mg/kg) intramuscular every 24 h for three days following the procedures (Fahmy et al. 2017). Periapical radiographs were used for apical closure confirmation.

Access cavities in the selected teeth in groups I and II were performed using round burs at high speed with water coolant, then by using a size 40 sterile file, the pulp was disturbed. Part of the supra-gingival plaque was collected and mixed with saline then inserted into pulp chambers by a sterile sponge saturated by the plaque suspension (Tawfik et al. 2013). After 1-month, the periapical radiograph was used to confirm the periapical lesion development.

### Procedural steps

#### Disinfection step

After 1 month, teeth surfaces were wiped with 0.12% chlorhexidine (Hexitol; Arab Drug Company, Cairo, Egypt) for disinfection and then isolated using cotton rolls, the previously selected teeth were re-accessed, and apical cementum was penetrated with #15 K-file then instrumented up to #60 K-file after electronic working length determination (Root ZX II; J. Morita, Kyoto, Japan). Final irrigation was performed by normal saline, 1.5% sodium hypochlorite (NaOCl) (Clorox Co, 10th of Ramadan, Egypt) (20 mL/canal for 5 min), and 17% ethylene diamine tetra acetic acid (EDTA) (PREVEST, DenPro. Jammu, India) (20 mL/canal) (Fahmy et al. 2017).

Equal portions of powdered ciprofloxacin (Ciprocin 500 mg; EPICO, Cairo, Egypt) and metronidazole (Flagel 500 mg; Aventis, Cairo, Egypt) were mixed with propylene glycol vehicle to a concentration of 1 mg/mL, to form Di-antibiotic paste, then applied up to cementoenamel junction (CEJ) in positive control group and study group for 1-month. Access cavities were sealed with glass ionomer cement (NOVA Glass-F; IMICRYL, Konya/ Turkey). After 1-month radiographic verification of periapical healing was performed (Endodontist 2016).

#### Revascularization step

Revascularization procedures were performed after 1-month according to American Association of Endodontists (AAE) guidelines, 2018. In the positive control group and study group, Di-antibiotic paste was copiously flushed from canals with 1.5% NaOCl, 20 ml of 17% EDTA solution (5 min/canal), and normal saline then dried with sterile paper points (Dia Dent, Chung Cheongbuk-do, Korea). Sterile hand files size #20–#35 K-files were used to apices over-instrumentation to allow blood flow to CEJ. Orifices were sealed with Mineral Trioxide Aggregates (MTA) (Rootdent, Tehno Dent, Russia), followed by a coronal seal with glass ionomer (Endodontist 2016).

**Fig. 2:**
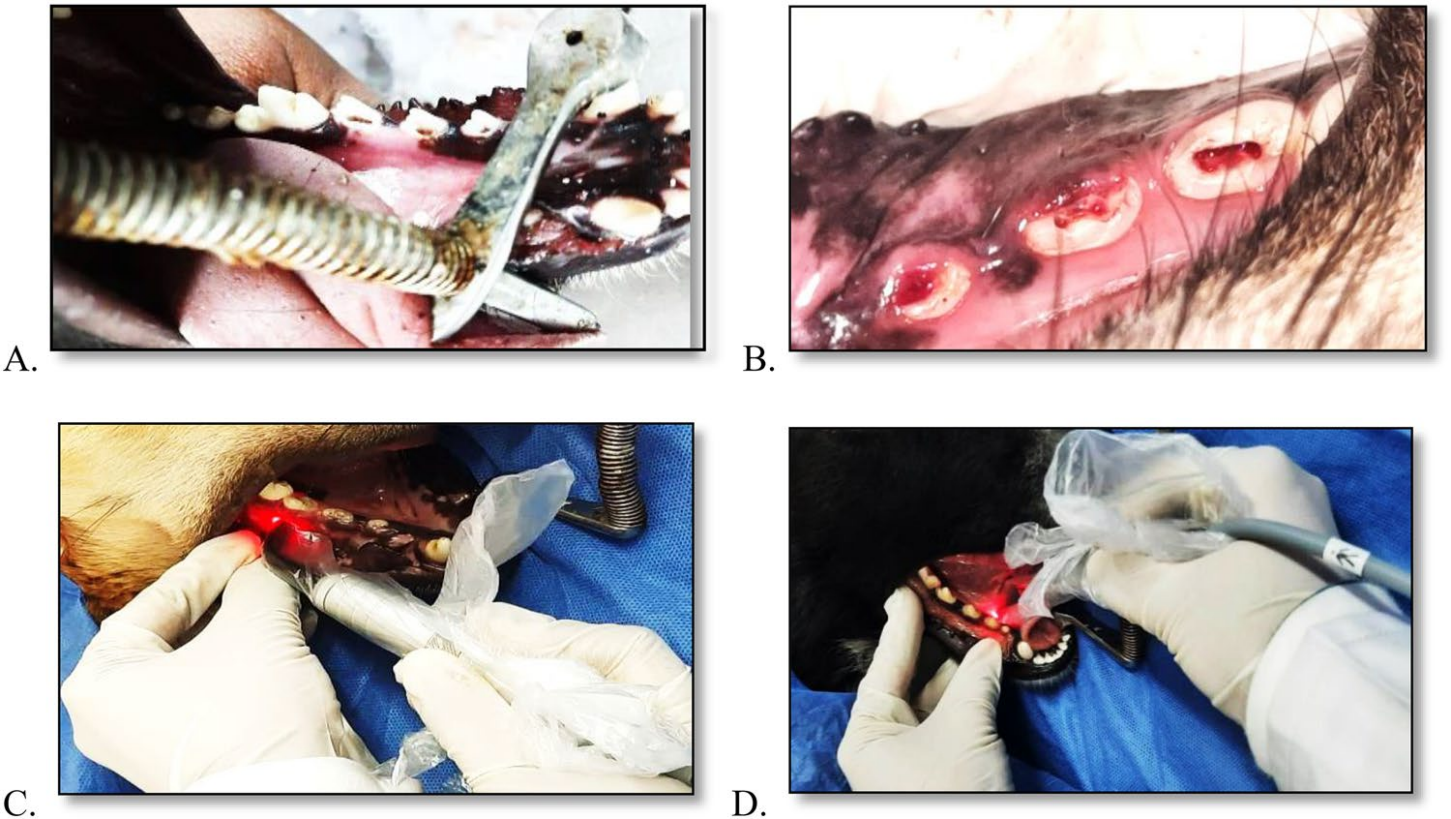
Access cavities (**A**), Blood clotting (**B**), Photobiomodulation therapy at the buccal side (**C**), And Photobiomodulation therapy at the lingual side (**D**)

Then the regenerative procedures in the study group were followed by photobiomodulation therapy on the apical root areas of the buccal and lingual surfaces at 48-h intervals for 2 weeks (6 sessions). Photobiomodulation was done by a diode semiconductor laser with 810-nm wavelength, Gallium- Aluminium –Arsenide laser (eleixxon_Claros_Pico, Germany), and output power of 300 mW. The laser beam was delivered with a biostimulation tip of 0.6 cm diameter in contact with the tissues, and the teeth were irradiated with a continuous wave emission of the laser for 4 s, energy (*E*) = 2.7 J and energy density of each application was 4 J/cm^2^ (Fekrazad et al. 2015). Fig. 2

All the teeth were monitored following therapy and the animals were sacrificed at different intervals (3 weeks, 10 weeks, and 15 weeks) and tissues were collected for histological evaluation.

### Sample preparation and histological evaluation

At each follow-up period, the animals were euthanized by an overdose of thiopental sodium 2 gm in 10 ml distilled water injected intravenously. The jaws were immediately dissected and the teeth with their surrounding structures were removed. Specimens were fixed in 4% paraformaldehyde (Formical, Decal Chemical Corporation, Congers, NY) for two days, then washed under tap water overnight, then decalcified in 10% EDTA (pH = 7.5) for three months, then embedded in paraffin for further preparation (Fahmy et al. 2017).

Each root was taken as an independent sample for histological evaluation. Sections were stained with hematoxylin and eosin (H&E) (Pascoe & Gatehouse 1986) and examined by the image analyzer computer system using the software Leica Qwin 500 (Germany) at Centre of Research and Dental Requirements, Faculty of Dentistry, Cairo University. The image analyzer was calibrated automatically to change the measurement units (pixels) into actual micrometre units. Neo-formed tissues were characterised by two blinded calibrated examiners. The vascular area per cent was measured in a measuring frame of 1,920,000 pixels, where five different fields for each subgroup slide were measured using a magnification (× 400).

Areas of the neovasculature staining were selected then the computer system converted the picture into a red binary colour that could be measured. The vascular area percentage was calculated for each field. Mean values of area per cent were obtained for each group for statistical evaluation. Fibroblasts were counted using the same software (the colour code threshold for nuclei of fibroblast cells was adjusted to exclude other undesired cells such as endothelial cells).

The variables (neovasculature and fibroblasts) were assessed using an image software by choosing the cell count for fibroblasts and measuring the surface area for new vessels. The software considered all the vessels in measuring surface area as we aimed to measure the revascularization of tissue involving measuring both blood and lymphatic vessels. Fibroblasts were identified based on their size, morphologies (spindle-shaped with a flat and oval nucleus), and their basophilic cytoplasm quite a lot of purplish blue staining with H&E stain and then were counted after setting the measurements of the software.

### Statistical analysis

Data were checked for normality using Shapiro–Wilk’s test. They were found to be normally distributed. Numerical data were presented as mean and standard deviation (SD) values and were analysed using repeated measure ANOVA for intergroup comparisons and repeated measures ANOVA followed by Bonferroni post hoc test for intragroup comparisons. The significance level was set at *p* < 0.05. Statistical analysis was performed with R statistical analysis software version 4.1.3 for Windows (R Core Team (2022).

## Results

Histological evaluation of the slides of the collected samples revealed that:

At different intervals of evaluation periods, samples of the negative control group showed normal pulp composed of loose connective tissue containing vessels, collagen fibres, fibroblasts, and odontoblasts (H&E X 200um).

At 3 weeks evaluation period, for positive control group, the photomicrograph showed less organised tissue formed of fibroblasts, collagen fibres, and vessels between homogenous dentine (X 50 um), as for the study group, the photomicrograph showed formation of a more organised pulp-like tissue than the positive control group; containing vessels, fibroblasts, and collagen fibres between homogenous dentine (H&E X 20 um).

In the 10th week of the evaluation period, the composed tissue was more organised and started to regenerate showing the formation of loose pulp-like tissue formed of vessels that were larger and dilated, and endothelial cells, and tissue were connected. Also, fibres were prominent in the study group (H&E X 20 um). For the positive control group, the photomicrograph showed the formation of pulp-like tissue between two dentinal walls formed of vessels, fibroblasts, and collagen fibres (H&E X100 um).

**Fig. 3:**
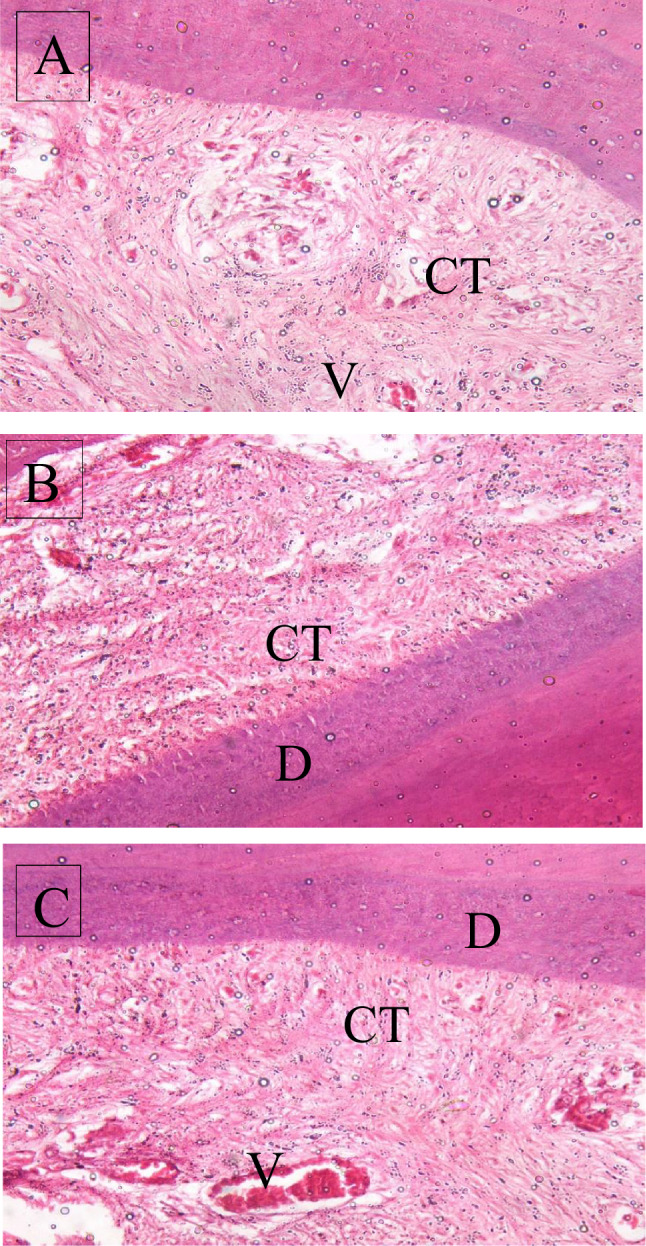
Photomicrograph showing **A** In-growth of loose connective tissue at the 3-week assessment of the study group. **B** Organization of the tissue and appearance of fibroblasts and narrow newly formed vascular capillaries at the 10-week assessment of the study group. **C**: Maturation of the tissue to show fibroblasts and numerous well-formed vascular capillaries at the 15-week assessment of the study group. *D* Dentin, *V* vascular area, *CT* connective tissue (H&E, 400X)

**Fig. 4:**
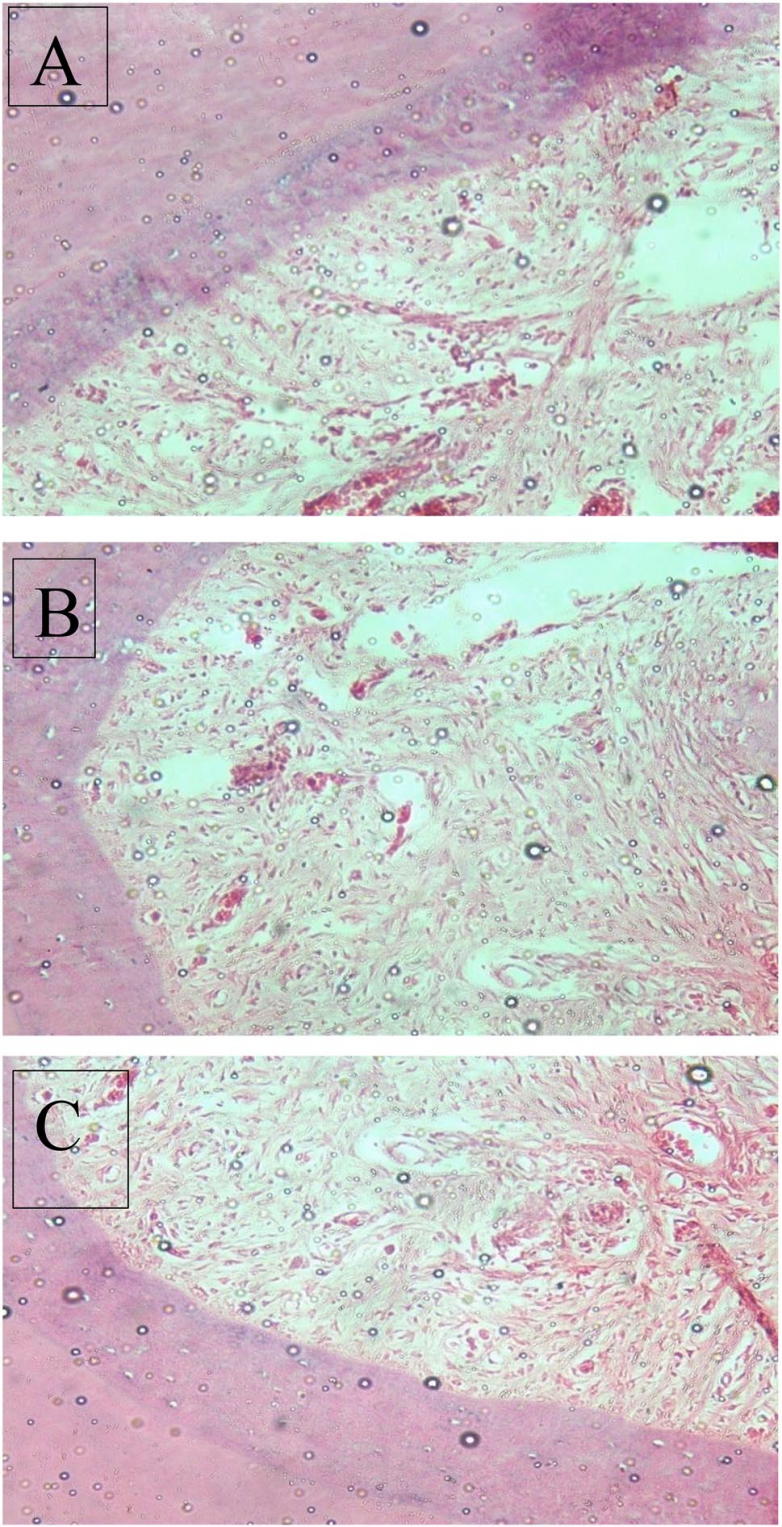
Photomicrograph showing **A** formation of granulation tissue devoid of pulp characteristics at the 3-week assessment of the positive control group. **B** 10-week assessment of positive control group. **C** 15-week assessment of positive control group. (H&E, 400X)

During 15 week evaluation period, the composed tissue was more organised, with numerous dilated vessels that were close to each other numerous fibroblasts, odontoblasts, collagen fibres, and newly formed circular capillaries, and an abundance of cells and vascularization between homogenous dentine (H&E X 50 um). For the positive control group, the photomicrograph showed the formation of fibroblasts, collagen fibres, vessels, and homogenous dentine (H&E X 100 um). (Figs. 3, 4).

### Vascular area percentage

At 3 weeks, there was an insignificant increase for the positive control group and the study group, with mean values, were 1.18 ± 0.50 and 1.68 ± 0.41 respectively, however, both groups were significantly lower than the negative control group (10.97 ± 3.07), (*p* < 0.001).

At 10 weeks, there was an insignificant increase for the positive control group and the study group, with mean values, were 1.29 ± 0.42 and 4.21 ± 0.55, respectively, however, both groups were significantly lower than the negative control group (11.79 ± 2.30) with *p*-value < 0.001.

At 15 weeks, there was an insignificant difference between the study group and the negative control group, with mean values was 9.76 ± 2.25 and 11.97 ± 2.37, respectively. However, both values were significantly higher than the positive control group (2.47 ± 1.0), (*p* < 0.001).

- For the positive control group, there was no statistically significant difference between the mean values at different intervals (*p* = 0.112). The highest mean value was recorded at 15 weeks (2.47 ± 1.00)
- For the study group, there was a significant difference between the mean values at different intervals (*p* < 0.001). The highest value was recorded at 15 weeks (9.76 ± 2.25)
- For the negative control group, there was no significant difference between the mean values at different intervals. (*p*<0.05). (Table 1)

**Table 1:** Mean and standard deviation (SD) values of vascular area percentage amongst studied groups

Interval	Area percentage (mean ± SD)			*p* value
	Positive control	Study	Negative control	
3 weeks	1.18 ± 0.50^Ba^	1.68 ± 0.41^Bb^	10.97 ± 3.07^Aa^	< 0.001*
10 weeks	1.29 ± 0.42^Ba^	4.21 ± 0.55^Bb^	11.79 ± 2.30^Aa^	< 0.001*
15 weeks	2.47 ± 1.00^Ba^	9.76 ± 2.25^Aa^	11.97 ± 2.37^Aa^	< 0.001*
*p* value	**0.112ns**	< 0.001**	** > 0.05ns**	

Intergroup comparisons were done using one way ANOVA, while intragroup comparisons were made using Repeated measure ANOVA. Means with different upper and lowercase superscript letters within the same horizontal row and vertical column respectively are significantly different

*ns* non-significant (*p* > 0.05)

*significant (*p* ≤ 0.05), One way ANOVA. **significant (p  ≤ 0.05), repeated measure ANOVA.

There was a significantly lower mean of vascular area percentages in positive control and study groups compared to negative control groups at 3 weeks.

### Fibroblasts count

At 3 weeks, there were insignificant differences in the study group and negative control group, where the mean values were 47.60 ± 11.28 and 43.98 ± 17.69, respectively. However, both values were significantly higher (*p* = 0.005) than the positive control group (20.80 ± 2.28).

At 10 weeks, there were no significant differences between different groups, where the mean values were 48.40 ± 5.50, 44.64 ± 17.05 and 35.80 ± 5.07 for the study group, the negative control group, and the positive control group, respectively (*p* = 0.126).

At 15 weeks, there were insignificant differences for the positive control group and the negative control group, where the mean values were 41.40 ± 1.14 and 44.67 ± 17.04, respectively. However, both values were significantly lower than the study group (56.60 ± 6.11) (*p* = 0.047).

- For the positive control group, there was a significant difference between the mean values measured at different intervals (*p* < 0.001). The highest mean value was measured at 15 weeks (41.40 ± 1.14).
- For the study group, there was no significant difference between mean values measured at different intervals (*p* = 0.191), with the highest mean value recorded at 15 weeks (56.60 ± 6.11).
- Whilst the negative control group showed an insignificant difference between the mean values at different intervals (*p* < 0.05). (Table 2)

**Table 2:** Mean and standard deviation (SD) values of fibroblast count amongst studied groups at different intervals

Interval	Fibroblasts count(mean ± SD)			*p* value
	Positive control	Study	Negative control	
3 weeks	20.80 ± 2.28^Bc^	47.60 ± 11.28^Aa^	43.98 ± 17.69^Aa^	0.005*
10 weeks	35.80 ± 5.07^Ab^	48.40 ± 5.50^Aa^	44.64 ± 17.05^Aa^	**0.126ns**
15 weeks	41.40 ± 1.14^Ba^	56.60 ± 6.11^Aa^	44.67 ± 17.04^ABa^	0.047*
*p* value	< 0.001**	**0.191ns**	** > 0.05ns**	

Intergroup comparisons were done using one way ANOVA, while intragroup comparisons were made using Repeated measure ANOVA.Means with different upper and lowercase superscript letters within the same horizontal row and vertical column, respectively are significantly different

*ns* non-significant (*p* > 0.05)

*significant (*p* ≤ 0.05), One way ANOVA. **significant (p  ≤ 0.05), repeated measure ANOVA.

## Discussion

Although clinical REPs have been centred on immature necrotic teeth since they are expected to have a more prominent possibility of pulp tissue regeneration, regenerative endodontic therapy in mature permanent teeth was supported by many studies as a practical treatment choice in mature permanent teeth with non-vital pulp and periapical pathosis (Arslan et al. 2019; El-Kateb et al. 2020; Glynis et al. 2021).

Also, in a recent histological study by Arslan et al. 2019, it was revealed that there is ingrowth of vital tissue inside the root canal system after REPs in mature permanent teeth and the structure of regenerated tissue was a mixture of a bone-like substance and fibrous connective tissue combined with vascular-like tissue, that are comparable to those recognised inside the root canals of immature teeth after REPs). Moreover, case reports of REPs in mature permanent teeth have presented promising outcomes in the resolution of signs and symptoms of periapical periodontitis (Saoud et al. 2016, 2014). Therefore mature permanent teeth were selected for the present study.

Dogs were selected as the animal model; since they have been presented as a proper model for experimental endodontics as they have large enough sized teeth, making endodontic treatments easier. In addition, dogs have dental tissue structures, healthy and diseased states, and apical repair comparable with humans, allowing the reproduction of near-to-real clinical situations. This happens in a shorter duration (average of 1/6 of humans) due to their faster growth rate (Katie Grzyb 2019). For such reasons, they are one of the most frequently used animal species in regenerative endodontics. Moreover, the premolar teeth were chosen due to their similarity to human molars. The selected dogs were 10–12 months old as the root apices of the premolar teeth were completed at 8 months of age (Nokhbatolfoghahaei et al. 2019).

Induction of periapical infection was done in positive control and study groups and confirmed with radiographic examination to simulate clinical conditions. The negative control group was not infected or received any intervention to avoid animal suffering during the evaluation period and to allow evaluation of normal dog’s pulp to be compared with regenerated pulp.

During REPs, 1.5% NaOCl for 5 min was used for root canal irrigation using an irrigating needle with a closed end and side vents positioned about 1 mm coronally from the root end to minimise cytotoxicity to the stem cells in the apical tissues (Endodontist, 2016). 17% EDTA (1 mL/canal, 1 min) irrigant was used as it stimulates the release of growth factors contained in the dentine matrix (Galler et al. 2015). Finally, all access cavities were sealed with Glass Ionomer (GI) as it has long-term sealing ability due to its electrochemical bonding ability to tooth structure (Sampathkumar et al. 2013).

A diode laser (810 nm) was selected for application in the study group in an attempt to find a supplementary technique that could enhance the biological functions of stem cells in the formed blood clot and structure of regenerated tissue in REPs that could lead to favourable outcomes, in addition, it is a portable and inexpensive device. An energy density of 2–4 J/cm^2^ was recommended from previous studies when used directly on teeth or indirectly above the apex (Paschoal and Santos-Pinto 2012 Sep 1; Toomarian et al. 2012). Also, 4 J/cm2 energy density for 48-h intervals was suggested to prevent the accumulated dose of photobiomodulation which can result in the bio-inhibition range (Fekrazad et al. 2015).

Also, a Diode laser with an infrared wavelength (700–1000 nm) is preferred to treat deeper tissues as red wavelengths penetrate 0.5 to 1 mm only but near-infrared energy penetrates 2 mm before losing 37% of its intensity (Padalkar and Pleshko 2015). Also, it can pass the surrounding tissues of the teeth and reach dental pulp cells, in addition, the energy of such lasers has a low absorption coefficient in haemoglobin and water (Rathod et al. 2022). Moreover, the optical probe was applied in close contact with the tissues to minimise light reflection from the surface of the tissue and increase the number of light photons that penetrate the tissues to reach the target tissue (Ng et al. 2018).

In addition, studies have shown that PBMT can improve cell migration, raise cell metabolism, and support the synthesis of growth factors, which, in turn, will enhance cell differentiation, and proliferation (Peng et al. 2017; Marques et al. 2017; Diniz et al. 2018) and could accelerate dentine regeneration after pulp exposure and improve dent alveolar-derived mesenchymal stem cells viability and proliferation, so it can enhance the endodontic regeneration process (Zaccara et al. 2019). Therefore, we suggested that a combination of PBMT with a blood clot scaffold could improve the regenerative procedures.

Histological assessment of the collected samples slides showed a more organised structure of the regenerated tissue at different intervals in the study group than the positive control group, in addition, the statistical analysis of the data regarding the vascular area percentage and fibroblasts count revealed higher mean values in the study group than positive control group. These findings could be attributed to the effect of PBMT which improves the environmental situations that enhance the survival rate of stem cells and prevent their premature death (Peng et al. 2017; Marques et al. 2017; Diniz et al. 2018).

Although the blood clot scaffold that is rich in stem cells was formed in the root canal in the same way in both the positive control group and the study group, the differences in the results may be related to the survival, and differentiation capacity of such cells. It has been suggested that such cells maintain their viability long enough after exposure to PBMT to allow tissue formation. Similar to the study observations, Tuby et al. 2011 discussed comparable findings in their research on the infarcted rat hearts, which revealed that although the number of stem cells in the infarcted area was elevated owing to homing of bone marrow stem cells into the infarcted area but the heart scarring progression was not decreased. Only rats whose bone marrow was exposed to laser irradiation had a prominent reduction in the size of their infarcted area. The stem cells were much more proliferative in irradiated bone marrow than the non-irradiated bone marrow.

Evaluating the tissue formed inside the root canals of the study group of the present study, revealed dental pulp-like characteristics in view of the formation of dilated new vessels, numerous fibroblasts, collagen fibres, and newly formed circular capillaries, and few odontoblasts. Such tissue has a quality that differs from those formed in other studies that were more comparable to bone or dental cement or osteoid tissue than to dental pulp-like structure (Torabinejad et al. 2014; Wang et al. 2015; Minic et al. 2022)^47–49^. However, our findings are comparable to those reported by Moreira et al. 2017 who revealed that blood clot scaffold combined with PBMT protocol resulted in a dental pulp-like tissue with vessels, odontoblast-like cell layer, and perivascular SCs.

On the other hand, these results were inconsistent with the study conducted by Fahmy et al. 2017 which revealed reduced cellular viability and vascular abundance. This difference could be attributed to their use of rapidly degrading collagen scaffolds in revascularization procedures. Further to this, collagen’s physical state had a dramatic effect on the degradation rate and provoked a different foreign body reaction. It could also be due to the combined therapy of PBM to REPs in our study.

Applying PBM therapy was suggested to prevent premature cell death. This could be partially related to a prominent increase in ATP, ROS content, angiogenesis, heat shock proteins, and inducible nitric oxide (NO) synthase (Rahmati et al. 2022). These biological effects of PBM therapy led us to apply this treatment to maintain the viability of stem cells present in a blood clot. Thus, one possible explanation for the dental pulp-like tissue regeneration in this study is the influence of PBM therapy, which allowed the stem cells to survive long enough to be affected by the growth factors in the blood clot released from the root canal walls. Other possible factors contributing to tissue regeneration are the vascular endothelial growth factor, expressed by different cell types and acts on the regulation of intercellular signals, and the process of angiogenesis, the formation of new vessels from pre-existing vessels (Grando Mattuella et al. 2007).

Supporting the present study findings, Arany et al. 2014 demonstrated that the use of PBMT stimulates endogenous bioactive factors that guide the differentiation of host stem cells to encourage the partially removed dental pulp regeneration process.

Recently, fibroblasts gained importance in pulp repair because they release growth factors that differentiate and direct stem cells to the injury site in addition to odontoblasts and pulp stem cells, pulp fibroblasts should be considered as a central cell that represents a real target in strategies to induce the dentin-pulp regeneration process (Jeanneau et al. 2017; Sequeira et al. 2021; Li et al. 2024). In our study, the mean values for fibroblast count were significantly higher in the study group than the positive control, and comparable to negative control at the 15th week period (*p* > 0.05) suggesting a possible increase at longer follow-up intervals. Jeanneau et al. 2017, reported insignificant differences in fibroblast proliferation and viability positive control group and negative control group. However, both groups were significantly lower than the study group, they observed an abundance of fibroblasts in the study group. This difference would be due to the energy density PBMT. An energy destiny of 4 J/cm2 was utilised in the present study which presented significantly higher values of cellular proliferation in view of vascularity and fibroblast proliferation. Also, PBMT influenced primary photochemical and photophysical events on the mitochondria leading to an increase in ATP and cell viability due to the change in the redox state towards oxidation. A secondary PBM effect occurs due to the modulation of biochemical reactions and changes in the redox state leading to DNA synthesis and consequently an increase in cell proliferation (AlGhamdi et al. 2012). This is supported by Bergamo et al. 2021***,*** who revealed that energy densities between 2.5 and 6.2 J/cm2 exhibited greater viability and proliferation of human-pulp fibroblasts.

Results of the current study are in agreement with other studies which revealed that the histological examination of human primary fibroblasts proliferation and viability when exposed to higher powers density of lasers at shorter time exhibit greater viability and gives the assumption that this laser application dosimetry is better for in vitro cell growth (Eduardo et al. 2008; Marques et al. 2017). One explanation could be the transient heating of the chromophores that might occur during longer periods and would trigger enzyme inhibition (Marques et al. 2017; Karu 2008).

Therefore, the main results showed that PBM therapy supports REPs in non-vital mature permanent teeth, and thus the authors of the current study rejected the null hypothesis.

The present study is one of few studies that investigated the effect of PBMT on the regeneration process of non-vital mature permanent teeth and the nature of regenerated tissues, and it would provide a promising and relevant strategy on how non-thermal and non-invasive photobiomodulation treatment impacts the capability of non-vital mature permanent teeth to regenerate or revitalise. The study also adopted rigorous measures to achieve a maximum level of standardisation such as the regenerative procedures were performed by a single investigator, assessment was carried out by expert blind calibrated assessors and the statistician was blinded about study groups.

However, some concerns must be taken into account; the nature of the trial that lacks the typical mimicking of the oral environment as well as differences in biological response between animal models and humans, also a possible biological differences in the regeneration response across the selected dogs In addition, the difficulties encountered in creating bleeding in canals of mature permanent teeth. Moreover, the examination of the longitudinal section of the pulp was difficult because of the microtone cutting of the samples. Moreover, fibroblast cells were evaluated based on their morphology, no cellular labelling or markers were used which cannot ascertain that the counted cells were purely fibroblasts due to their morphological similarity to other cell types.

## Conclusions

Within the limitations of the current study and according to the results, the following may be concluded:

- Revascularization of non-vital mature permanent teeth can be a successful alternative treatment modality for regaining vascularity.
- PBM after blood clot revascularization was found to be an effective way to improve the structure of regenerated tissue.
- The positive results recorded by the end of follow-up interval would suggest future studies with longer follow periods to understand the long-term process of regeneration. In addition, further research focussing on characterising regenerated tissues would be promising

## Data Availability

The datasets used during the current study are available from the corresponding author upon reasonable request. All data analysed during this study are included in this published article in the form of tables and figures.

## Abbreviations

PBM: Photobiomodulation
MTA: Mineral trioxide aggregates
EDTA: Ethylene diamine tetraacetic acid
NaOCl: Sodium hypochlorite
MSC: Mesenchymal stem cells
CEJ: Cementoenamel junction
GI: Glass ionomer
H&E: Hematoxylin and eosin
SD: Standard deviation
SCAPs: Stem cells of apical papilla
REPs: Regenerative endodontic procedures

## References

[CR2] ∙ AlGhamdi KM, Kumar A, Moussa NA. Low-level laser therapy: a useful technique for enhancing the proliferation of various cultured cells. Lasers Med Sci. 2012. 10.1007/s10103-011-0885-2. 21274733 10.1007/s10103-011-0885-2

[CR3] ∙ Alghofaily M, Torabinejad M, Nosrat A. Regenerative endodontic treatment using periapical blood or circulating blood as scaffold: a volumetric analysis. J Endod. 2022;48(5):625–31. 10.1016/j.joen.2022.01.008. 35218760 10.1016/j.joen.2022.01.008

[CR4] Alnagar AM, Mahmoud M, Gutknecht N, Moreira MS, Sarra G, Carvalho GL, et al. Effect of photobiomodulation therapy on regenerative endodontic procedures: a scoping review. Lasers Dent Sci. 2019. 10.1007/s41547-019-00076-5.

[CR5] Al-Nazhan S, Al-Sulaiman A, Al-Rasheed F, Alnajjar F, Al-Abdulwahab B, Al-Badah A. Microorganism penetration in dentinal tubules of instrumented and retreated root canal walls. Restor Dent Endod. 2014;39(4):258. 10.5395/rde.2014.39.4.258. 25383343 10.5395/rde.2014.39.4.258 PMC4223094

[CR6] Arany PR, Cho A, Hunt TD, Sidhu G, Shin K, Hahm E, et al. Photoactivation of endogenous latent transforming growth factor-β1 directs dental stem cell differentiation for regeneration [Internet]. Sci Transl Med. 2014;6(238):238ra69. 10.1126/scitranslmed.3008234. 24871130 10.1126/scitranslmed.3008234 PMC4113395

[CR7] Arapostathis K, Velonis D, Chala M. Laser-Assisted Pediatric Dentistry. In: Arapostathis K, Velonis D, Chala M, (Eds) Springer, Cham; 2023. p. 339–76. Available from: https://link.springer.com/10.1007/978-3-031-43338-2_11

[CR8] Arslan H, Ahmed HMA, Şahin Y, Doğanay Yıldız E, Gündoğdu EC, Güven Y, et al. Regenerative endodontic procedures in necrotic mature teeth with periapical radiolucencies: a preliminary randomized clinical study. J Endod. 2019;45(7):863–72. 10.1016/j.joen.2019.04.005. 31155298 10.1016/j.joen.2019.04.005

[CR9] Bergamo MT, Vitor LLR, Dionísio TJ, Marques NCT, Oliveira RC, Ambrosio ECP, et al. Could the photobiomodulation therapy induce angiogenic growth factors expression from dental pulp cells? Lasers Med Sci. 2021;36(8):1751–8. 10.1007/s10103-021-03291-4. 33796964 10.1007/s10103-021-03291-4

[CR10] Bhuva B, Ikram O. Complications in endodontics. Prim Dent J NLM (Medline). 2020;9(4):52–8. 10.1177/2050168420963306. 10.1177/2050168420963306 33225854

[CR11] Borisova-Papancheva T, Svetlozarova S, Kostadinova N. Causes of persistent apical periodontitis after endodontic treatment. Varna Med Forum. 2022;11(1):186.

[CR12] Chrepa V, Henry MA, Daniel BJ, Diogenes A. Delivery of apical mesenchymal stem cells into root canals of mature teeth. J Dent Res. 2015;94(12):1653–9. 10.1177/0022034515596527. 26195498 10.1177/0022034515596527 PMC6728573

[CR13] Cronshaw M, Mylona V. Photobiomodulation therapy within clinical dentistry: theoretical and applied concepts. 2023. p. 173–236. 10.1007/978-3-031-43338-2_7.

[CR14] Diniz IMA, Carreira ACO, Sipert CR, Uehara CM, Moreira MSN, Freire L, et al. Photobiomodulation of mesenchymal stem cells encapsulated in an injectable rhBMP4-loaded hydrogel directs hard tissue bioengineering. J Cell Physiol. 2018;233(6):4907–18. 10.1002/jcp.26309. 29215714 10.1002/jcp.26309

[CR15] Dipalma G, Inchingolo AM, Patano A, Palumbo I, Guglielmo M, Trilli I, et al. Photobiomodulation and growth factors in dentistry : a systematic review. Photonics. 2023. 10.3390/photonics10101095.

[CR16] Dissanayaka WL, Zhu L, Hargreaves KM, Jin L, Zhang C. In vitro analysis of scaffold-free prevascularized microtissue spheroids containing human dental pulp cells and endothelial cells. J Endod. 2015;41(5):663–70. 10.1016/j.joen.2014.12.017. 25687363 10.1016/j.joen.2014.12.017

[CR17] ∙ Eduarde FDP, Bueno DF, De Freitas PM, Marques MM, Passos-Bueno MR, Eduarde CDP, et al. Stem cell proliferation under low intensity laser irradiation: a preliminary study. Lasers Surg Med. 2008;40(6):433–8. 10.1002/lsm.20646. 18649378 10.1002/lsm.20646

[CR18] El-Kateb NM, El-Backly RN, Amin WM, Abdalla AM. Quantitative assessment of intracanal regenerated tissues after regenerative endodontic procedures in mature teeth using magnetic resonance imaging: a randomized controlled clinical trial. J Endod. 2020;46(5):563–74. 10.1016/j.joen.2020.01.026. 32173020 10.1016/j.joen.2020.01.026

[CR1] Endodontist AAF (2016) AAE Clinical considerations for a regenerative procedure.

[CR19] Fahmy SH, Hassanien EES, Nagy MM, El Batouty KM, Mekhemar M, Fawzy El Sayed K, et al. Investigation of the regenerative potential of necrotic mature teeth following different revascularisation protocols. Aust Endod J. 2017;43(2):75–84. 10.1111/aej.12210. 10.1111/aej.12210 28766808

[CR20] Farhad A, Saatchi M, Bagherieh S. Effect of citric acid versus EDTA on radiographic root development in regenerative endodontic treatment: an animal study. J Endod. 2022;48(4):535–41. 10.1016/j.joen.2022.01.001.Epub2022. 35026229 10.1016/j.joen.2022.01.001

[CR21] Faul F, Erdfelder E, Lang A-G, Buchner A. G*Power 3: A flexible statistical power analysis program for the social, behavioral, and biomedical sciences. Behav Res Methods. 2007;39(2):175–91. 10.3758/BF03193146. 17695343 10.3758/bf03193146

[CR22] Fekrazad R, Seraj B, Ghadimi S, Dehghan MM. The effect of low-level laser therapy (810 nm) on root development of immature permanent teeth in dogs. Lasers Med Sci. 2015;30(4):1251–7. 10.1007/s10103-014-1588-2. 24858234 10.1007/s10103-014-1588-2

[CR23] Fenwick N, Danielson P, Griffin G. Survey of Canadian animal-based researchers’ views on the three Rs: replacement, reduction and refinement. PLoS ONE. 2011. 10.1371/journal.pone.0022478. (**Epub 2011 Aug 17**). 21857928 10.1371/journal.pone.0022478 PMC3157340

[CR24] Galler KM, Buchalla W, Hiller KA, Federlin M, Eidt A, Schiefersteiner M, Schmalz GJ. Influence of root canal disinfectants on growth factor release from dentin. J Endod. 2015;41(3):363–8. 10.1016/j.joen.2014.11.021. (**Epub 2015 Jan 13**). 25595468 10.1016/j.joen.2014.11.021

[CR26] Glynis A, Foschi F, Kefalou I, Koletsi D, Tzanetakis GN. Regenerative endodontic procedures for the treatment of necrotic mature teeth with apical periodontitis: a systematic review and meta-analysis of randomized controlled trials. J Endod. 2021;47(6):873–82. 10.1016/j.joen.2021.03.015. (**Epub 2021 Ma**). 33811981 10.1016/j.joen.2021.03.015

[CR27] Goldberg M. Root canal treatment (RCT): From traditional endodontic therapies to innovating pulp regeneration [Internet]. J Dent Oral Disord Ther. 2016;4(2):1–6.

[CR28] Grando Mattuella L, Westphalen Bento L, Poli de Figueiredo JA, Eduardo Nör J, Borba de Araujo F, Christina Medeiros Fossati A. Vascular endothelial growth factor and its relationship with the dental pulp. J Endod. 2007;33(5):524–30. 10.1016/j.joen.2007.01.003. 17437865 10.1016/j.joen.2007.01.003

[CR29] Hamblin MR. Mechanisms and applications of the anti-inflammatory effects of photobiomodulation. AIMS Biophys. 2017;4(3):337–61. 10.3934/biophy.2017.3.337. 28748217 10.3934/biophy.2017.3.337 PMC5523874

[CR30] He L, Zhong J, Gong Q, Kim SG, Zeichner SJ, Xiang L, et al. Treatment of necrotic teeth by apical revascularization: meta-analysis. Sci Rep. 2017. 10.1038/s41598-017-14412-x. 29066844 10.1038/s41598-017-14412-x PMC5655000

[CR31] Jeanneau C, Lundy FT, El Karim IA, About I. Potential therapeutic strategy of targeting pulp fibroblasts in dentin-pulp regeneration. J Endod. 2017;43(9):S17-24. 10.1016/j.joen.2017.06.007. 28778507 10.1016/j.joen.2017.06.007

[CR32] Johns DA, Shivashankar VY, Krishnamma S, Johns M. Use of photoactivated disinfection and platelet-rich fibrin in regenerative endodontics. J Conserv Dent. 2014;17(5):487–90. 10.4103/0972-0707.139850. 25298655 10.4103/0972-0707.139850 PMC4174714

[CR33] Kahler B, Rossi-Fedele G, Chugal N, Lin LM. An evidence-based review of the efficacy of treatment approaches for immature permanent teeth with pulp necrosis. J Endod. 2017;43(7):1052–7. 10.1016/j.joen.2017.03.003. 28511779 10.1016/j.joen.2017.03.003

[CR34] Karamifar K, Tondari A, Saghiri MA. Endodontic periapical lesion: an overview on the etiology, diagnosis and current treatment modalities. Eur Endod J. 2020;5(2):54–67. 10.14744/eej.2020.42714. 32766513 10.14744/eej.2020.42714 PMC7398993

[CR35] Karu TI. Mitochondrial signaling in mammalian cells activated by red and near-IR radiation. Photochem Photobiol. 2008;84(5):1091–9. 10.1111/j.1751-1097.2008.00394.x. 18651871 10.1111/j.1751-1097.2008.00394.x

[CR36] Katie Grzyb D. 5 interesting facts about your dog’s teeth [Internet]. 2019. Available from: http://www.petmed.com/dog/general-health/5-interesting-facts-about-your-dogs-teeth

[CR38] Laureys WGM, Cuvelier CA, Dermaut LR, De Pauw GAM. The critical apical diameter to obtain regeneration of the pulp tissue after tooth transplantation, replantation, or regenerative endodontic treatment. J Endod. 2013;39(6):759–63. 10.1016/j.joen.2013.02.004. 23683275 10.1016/j.joen.2013.02.004

[CR40] Li XL, Fan W, Fan B. Dental pulp regeneration strategies: a review of status quo and recent advances. Bioact- Mater. 2024;38:258–75. 10.1016/j.bioactmat.2024.04.031. 38745589 10.1016/j.bioactmat.2024.04.031 PMC11090883

[CR41] Lovelace TW, Henry MA, Hargreaves KM, Diogenes A. Evaluation of the delivery of mesenchymal stem cells into the root canal space of necrotic immature teeth after clinical regenerative endodontic procedure. J Endod. 2011;37(2):133–8. 10.1016/j.joen.2010.10.009. 21238791 10.1016/j.joen.2010.10.009

[CR42] Marques MM, de Cara SPHM, Abe GL, Pedroni ACF, Diniz IMA, Moreira MS. Effects of photobiomodulation therapy in dentoalveolar-derived mesenchymal stem cells: a review of literature. Lasers Dent Sci. 2017. 10.1007/s41547-017-0002-3.

[CR43] Minic S, Vital S, Chaussain C, Boukpessi T, Mangione F. Tissue characteristics in endodontic regeneration a systematic review. Int J Mol Sci I. 2022;23(18):10534. 10.3390/ijms231810534. 10.3390/ijms231810534 PMC9504778 36142446

[CR44] Moreira MS, Diniz IM, Rodrigues MFSD, de Carvalho RA, de Almeida Carrer FC, Neves II, et al. In vivo experimental model of orthotopic dental pulp regeneration under the influence of photobiomodulation therapy. J Photochem Photobiol B Biol. 2017;1(166):180–6. 10.1016/j.jphotobiol.2016.11.022. 10.1016/j.jphotobiol.2016.11.022 27927605

[CR46] Ng DY, Chan AK, Dalci O, Petocz P, Papadopoulou AK, Darendeliler MA. A pilot study of laser energy transmission through bone and gingiva. J Am Dent Assoc. 2018;149(8):704–11. 10.1016/j.adaj.2018.04.002. 29935726 10.1016/j.adaj.2018.04.002

[CR47] Nokhbatolfoghahaei H, Paknejad Z, Bohlouli M, Rad MR, Khojasteh A. Animal models in dental research. Appl Biomed Eng Dent. 2019. 10.1007/978-3-030-21583-5_18.

[CR48] Padalkar MV, Pleshko N. Wavelength-dependent penetration depth of near infrared radiation into cartilage. Anal R Soc Chem. 2015;140(7):2093–100. 10.1039/C4AN01987C. 10.1039/c4an01987c PMC4418489 25630381

[CR49] Paschoal MAB, Santos-Pinto L. Therapeutic effects of low-level laser therapy after premolar extraction in adolescents: a randomized double-blind clinical trial. Photomed Laser Surg. 2012;30(9):559–64. 10.1089/pho.2012.3243. 22870960 10.1089/pho.2012.3243

[CR50] Pascoe S, Gatehouse D. The use of a simple haematoxylin and eosin staining procedure todemonstrate micronuclei within rodent bone marrow. Mutat Res Mutagen Relat Subj. 1986;164(4):237–43. 10.1016/0165-1161(86)90057-9. 10.1016/0165-1161(86)90057-9 2427945

[CR51] Peng C, Zhao Y, Wang W, Yang Y, Qin M, Ge L. Histologic findings of a human immature revascularized/regenerated tooth with symptomatic irreversible pulpitis. J Endod. 2017;43(6):905. 10.1016/j.joen.2017.01.031. 28416306 10.1016/j.joen.2017.01.031

[CR52] R Core Team. R: a language and environment for statistical computing. R Foundation for statistical computing, Vienna, Austria. Sci. Res. Publ. [Internet]. 2022; Available from: https://www.r-project.org/

[CR53] Rahmati A, Abbasi R, Najafi R, Rezaei-soufi L, Karkehabadi H. Effect of diode low level laser and red light emitting diode irradiation on cell proliferation and osteogenic/odontogenic differentiation of stem cells from the apical papilla. BMC Oral Health. 2022;22(1):543. 10.1186/s12903-022-02574-8. 36434589 10.1186/s12903-022-02574-8 PMC9701043

[CR54] Rathod A, Jaiswal P, Bajaj P, Kale B, Masurkar D. Implementation of low-level laser therapy in dentistry: a review. Cureus. 2022;14(9): e28799. 10.7759/cureus.28799. 36225465 10.7759/cureus.28799 PMC9534528

[CR55] Saghiri MA, Asatourian A, Sorenson CM, Sheibani N. Role of angiogenesis in endodontics: contributions of stem cells and proangiogenic and antiangiogenic factors to dental pulp regeneration. J Endod. 2015;41(6):797–803. 10.1016/j.joen.2014.12.019. 25649306 10.1016/j.joen.2014.12.019 PMC5223201

[CR56] Sampathkumar SJ, Narasimiah SKB, Vadivel SP. Role of provisional restorations in endodontic therapy. J Pharm Bioallied Sci. 2013;5(Suppl 1):S120–4. 10.4103/0975-7406.113311. 23946564 10.4103/0975-7406.113311 PMC3722693

[CR57] Saoud TMA, Sigurdsson A, Rosenberg PA, Lin LM, Ricucci D. Treatment of a large cyst like inflammatory periapical lesion associated with mature necrotic teeth using regenerative endodontic therapy. J Endod. 2014;40(12):2081–6. 10.1016/j.joen.2014.07.027. 25292168 10.1016/j.joen.2014.07.027

[CR58] Saoud TM, Martin G, Chen YHM, Chen KL, Chen CA, Songtrakul K, et al. Treatment of mature permanent teeth with necrotic pulps and apical periodontitis using regenerative endodontic procedures: a case series. J Endod. 2016;42(1):57–65. 10.1016/j.joen.2015.09.015. 26525552 10.1016/j.joen.2015.09.015

[CR59] Sequeira DB, Oliveira AR, Seabra CM, Palma PJ, Ramos C, Figueiredo MH, et al. Regeneration of pulp-dentin complex using human stem cells of the apical papilla: in vivo interaction with two bioactive materials. Clin Oral Investig. 2021;25(9):5317–29. 10.1007/s00784-021-03840-9. 33630165 10.1007/s00784-021-03840-9

[CR60] Syed-Picard FN, Ray HL, Kumta PN, Sfeir C. Scaffoldless tissue-engineered dental pulp cell constructs for endodontic therapy. J Dent Res. 2014;93(3):250–5. 10.1177/0022034513517901. 24401375 10.1177/0022034513517901 PMC4239153

[CR61] Tawfik H, Abu-Seida AM, Hashem AA, Nagy MM. Regenerative potential following revascularization of immature permanent teeth with necrotic pulps. Int Endod J. 2013;46(10):910–22. 10.1111/iej.12079. 23480261 10.1111/iej.12079

[CR62] Tong HJ, Rajan S, Bhujel N, Kang J, Duggal M, Nazzal H. Regenerative endodontic therapy in the management of nonvital immature permanent teeth: a systematic review—outcome evaluation and meta-analysis. J Endod. 2017;43(9):1453–64. 10.1016/j.joen.2017.04.018. 28743431 10.1016/j.joen.2017.04.018

[CR63] Toomarian L, Fekrazad R, Tadayon N, Ramezani J, Tunér J. Stimulatory effect of low-level laser therapy on root development of rat molars: a preliminary study. Lasers Med Sci. 2012;27(3):537–42. 10.1007/s10103-011-0935-9. 21614480 10.1007/s10103-011-0935-9

[CR64] Torabinejad M, Faras H, Corr R, Wright KR, Shabahang S. Histologic examinations of teeth treated with 2 scaffolds: a pilot animal investigation. J Endod. 2014;40(4):515–20. 10.1016/j.joen.2013.12.025. 24666902 10.1016/j.joen.2013.12.025

[CR65] Torabinejad M, Nosrat A, Verma P, Udochukwu O. Regenerative endodontic treatment or mineral trioxide aggregate apical plug in teeth with necrotic pulps and open apices: a systematic review and meta-analysis. J Endod. 2017;43(11):1806–20. 10.1016/j.joen.2017.06.029. 28822564 10.1016/j.joen.2017.06.029

[CR66] Tuby H, Maltz L, Oron U. Induction of autologous mesenchymal stem cells in the bone marrow by low-level laser therapy has profound beneficial effects on the infarcted rat heart. Lasers Surg Med. 2011;43(5):401–9. 10.1002/lsm.21063. 21674545 10.1002/lsm.21063

[CR67] Wang Y, Zhu X, Zhang C. Pulp revascularization on permanent teeth with open apices in a middle-aged patient. J Endod. 2015;41(9):1571–5. 10.1016/j.joen.2015.04.022. 26071100 10.1016/j.joen.2015.04.022

[CR68] Zaccara IM, Jardine AP, Mestieri LB, Quintana RM, Jesus L, Moreira MS, et al. Influence of photobiomodulation therapy on root development of rat molars with open apex and pulp necrosis. Braz Oral Res. 2019;33:e084. 10.1590/1807-3107bor-2019.vol33.0084. 31460610 10.1590/1807-3107bor-2019.vol33.0084

